# Linguistic distances between native languages and Chinese influence acquisition of Chinese character, vocabulary, and grammar

**DOI:** 10.3389/fpsyg.2022.1083574

**Published:** 2023-01-13

**Authors:** Xingsan Chai, Jie Bao

**Affiliations:** Institute on Educational Policy and Evaluation of International Students, Beijing Language and Culture University, Beijing, China

**Keywords:** linguistic distance, Chinese proficiency, language knowledge, Chinese as a second language acquisition, cross-language transfer

## Abstract

How linguistic distance affects second language acquisition is a major concern in cross-language transfer research. However, no study has explored how systematic differences between Chinese and learners’ native language (L1) influences Chinese character, vocabulary, and grammar acquisition, or how these influences change as Chinese proficiency improves. To address this, we employed the World Atlas of Language Structures (WALS) index method to multidimensionally quantify the linguistic distance between Chinese and L1, and examined the effect of systematic linguistic distance on acquisition of Chinese character (Quasi-Experiment 1), vocabulary (Quasi-Experiment 2), and grammatical knowledge (Quasi-Experiment 3) in Chinese as a second language (CSL) learners with elementary, intermediate, and advanced Chinese proficiency levels. We examined a random sample of 58,240 CSL learners’ test scores from 24 different L1 backgrounds, and analyzed 2,250 CSL learners’ Chinese character, vocabulary, and grammar scores in each of the three quasi-experiments. We found that closer linguistic distance facilitated more favorable Chinese character and vocabulary acquisition at elementary, intermediate, and advanced Chinese proficiency levels, and that the influence of linguistic distance on CSL learners’ vocabulary acquisition tended to decrease as Chinese proficiency increased. Finally, linguistic difference did not significantly affect CSL learners’ grammar acquisition at elementary proficiency, but as Chinese proficiency improved, an L1 interference effect occurred among CSL learners with a short linguistic distance from Chinese, which hindered grammar acquisition. These results suggest that linguistic distance has differential proficiency-dependent effects on Chinese character, vocabulary, and grammar acquisition.

## 1. Introduction

The role of native language (L1) in second language acquisition is a core issue in cross-language transfer research (Odlin, 1989; Ellis, 1994). Cross-language transfer is the influence of a language acquired or learned earlier on the new learning or acquisition of another language (Jarvis and Pavlenko, 2008). In a positive cross-language transfer, the acquired language promotes target language acquisition; in a negative cross-language transfer, the acquired language hinders target language acquisition (Odlin, 2001). Structuralist linguistics and behaviorist psychology assert that greater similarity between L1 and a target language facilitates target language acquisition, whereas greater differences are detrimental to target language acquisition (Lado, 1957). However, several studies have found that L1’s influence on learners’ second language acquisition is limited by several factors (Odlin, 1989; Odlin and Jarvis, 2004; Jarvis and Pavlenko, 2008), and that the extent to which L1 influences target language acquisition is not necessarily the same for learners with different target language proficiency levels (Sjoholm, 1995; Jarvis, 1998; Cenoz, 2001; Ringbom, 2007). Currently, there is no consensus on L1’s role in second language acquisition.

Linguistic distance is an important indicator of L1 and second language similarity, and thus an important independent variable in cross-language transfer studies. Many previous studies on acquiring Chinese as a second language (CSL) examined linguistic distance effects on Chinese character (Jiang, 2003; Zhang, 2017; Deng and Hu, 2022), vocabulary (Hong, 2013; Xu, 2014; Hsieh and Wang, 2020; Chai and Ma, 2022; Tang and Chan, 2022), and grammar (Yuan, 1998, 2004; Wu and Zhao, 2018; Hao et al., 2022) acquisition. Chinese comprises several subsystems, such as Chinese character, vocabulary, and grammar, that interact across levels (De Saussure, 1959). However, previous studies focused on similarities and differences in specific features of learners’ L1 and Chinese linguistics, and neglected to examine the effect of systematic differences between learners’ L1 and Chinese on language knowledge acquisition (Hao et al., 2021). In addition, the validity of extending homogeneous sample-based laboratory studies’ findings to heterogeneous environments is controversial (Cui et al., 2018; Floccia et al., 2018). Therefore, it is of considerable theoretical and practical importance to study linguistic distance effects on Chinese character, vocabulary, and grammar acquisition using a large sample in a non-laboratory setting.

Multidimensional methods for quantifying linguistic distance have developed through an in-depth intersection of linguistics, statistics, computer science, and other disciplines. Numerous studies have examined the impact of linguistic distance on CSL acquisition under heterogeneous conditions based on language test data. However, the findings concentrate on acquisition of CSL language skills at the macro level, such as speaking, reading, writing, and listening (Wang and Cui, 2018). Both language knowledge and language skills are important components of language competence (Bachman, 1990), and language knowledge is the basis for developing language skills. Ideally, higher learner language skills proficiency reflects higher language knowledge proficiency. In practice, however, language skills and language knowledge development are not perfectly synchronized, because second language acquisition is influenced by several factors, such as knowledge characteristics within the language subsystem, learning difficulty, and learners’ second language proficiency (De Jong et al., 2013; Dai, 2014). Currently, no comprehensive empirical study of large-scale, standardized language test data has investigated the impact of systematic differences between L1 and Chinese on CSL learners’ Chinese character, vocabulary, and grammar acquisition.

Considering the contested findings in theoretical and empirical studies on L1’s role in second language acquisition, and that Chinese characters, vocabulary, and grammar differ from those of alphabetic languages, previous studies’ findings are unlikely to generalize to the role of L1 in CSL learners’ language knowledge acquisition.

Therefore, this study used the Hànyu Shuiˇpíng Kǎoshì (HSK; literally translated as Chinese Proficiency Test) to measure CSL learners’ language proficiency, and systematically investigated linguistic distance influence patterns on Chinese character, vocabulary, and grammar acquisition at elementary, intermediate, and advanced proficiency, using multidimensional linguistic distance quantification. This study provides systematic and persuasive evidence in relation to current theoretical debates on cross-language transfer, and sheds light on the study of CSL acquisition and teaching.

## 2. Literature review

### 2.1. Cross-language transfer theory

Cross-language transfer is a core issue in second language acquisition field (Klein et al., 1986; Gass and Selinker, 1992). Behaviorism, cognition, and social schools differ in both their theoretical claims and empirical research findings regarding whether and how L1 influences second language acquisition (Cook and Singleton, 2014).

Behaviorism asserts that L1 is the primary cause of learners’ language acquisition difficulties and errors, with a greater difference between L1 and the target language leading to more difficulty learning the target language (Lado, 1957; Stockwell et al., 1965). However, the behaviorist viewpoint raises theoretical and empirical questions. From a theoretical perspective, many researchers argue that behaviorist theories of cross-language transfer ignore learners’ subjectivity, while viewing language acquisition as a stimulus-reflection habit-forming process (Krashen, 1985; Swain and Lapkin, 1995; Long, 1996). From an empirical perspective, numerous research findings demonstrate that cross-language differences do not necessarily lead to second language acquisition difficulties (Grauberg, 1971; Dulay and Burt, 1973), or serve as a main reason for second language acquisition difficulties (Kleinmann, 1978; Zobl, 1983; Pica, 1994).

The cognitive school’s two main branches, universal grammar theory and connectionist framework theory, emphasize the learner’s role as a cognitive subject in language acquisition (Cook and Singleton, 2014). The universal grammar theory posits two distinct views on whether L1 affects second language acquisition. One holds that universal grammar covers the second language’s initial state, and that L1 has no effect on second language acquisition (Bley-Vroman, 1990; Epstein, 1996; Platzack, 1996), while the other holds that L1 differs from the initial state of the second language, and that L1 affects second language acquisition (Schwartz and Sprouse, 1996; Vainikka and Young-Scholten, 1996). Both positions are supported by empirical studies. Different from the universal grammar theory, which emphasizes innate determinism, the connectionist framework theory holds that second language acquisition difficulty and learning speed largely depend on target language input frequency (Goldschneider and DeKeyser, 2001; Ellis, 2006), where L1 plays a role in regulating learners’ absorption of second language input. If the second language’s input information is similar to that of L1, a positive cross-language transfer occurs; if the second language’s input information differs from that of L1, a negative cross-language transfer occurs because the second language’s output is not what is expected. More importantly, at the elementary proficiency level of second language acquisition, learners usually draw on their L1 knowledge to compensate because their knowledge of the second language is not sufficient to allow them to fully express themselves. Therefore, learners at an elementary proficiency level are more likely to be affected by their L1, which also explains why second language acquisition development shows asymptotic and dynamic characteristics (Cook and Singleton, 2014).

Unlike the cognitive school, which views language as a psychological phenomenon, the sociocultural school focuses on both the influence of learners’ elements on second language acquisition and on how social and cultural aspects affect second language acquisition (Vygotsky, 1978; Ellis, 2008; Green and Abutalebi, 2013; Tong and Yip, 2015). Vygotsky’s sociocultural theory is influential in this school (Lantolf and Appel, 1994; Lantolf and Thorne, 2006), and posits that learners’ L1 serves as a mediation tool that helps them achieve their communicative purposes when learning a second language. Numerous cross-language transfer studies based on sociocultural theory also show that L1’s influence on learners’ second language acquisition varies with their second language proficiency; when learners are not proficient enough to control cognitive activity in the second language, they rely more on their L1 to complete challenging mental tasks (Frawley and Lantolf, 1985; Swain and Lapkin, 1998; Centeno-Cortés and Jiménez Jiménez, 2004).

In summary, various schools of cross-language transfer theory agree that L1 plays a role in second language acquisition (MacWhinney and Kroll, 2005). Most scholars acknowledge that cross-language transfer is a complex and dynamic cognitive process that is influenced by a variety of factors, including linguistics, psychology, and society, rather than a mechanical habit-forming process based on stimulus–response (Xu Q. et al., 2013). Therefore, L1’s role in second language acquisition needs to be examined from a systematic and developmental perspective (Larsen-Freeman, 1997; Larsen-Freeman and Cameron, 2008).

### 2.2. Specificity of Chinese characters, vocabulary, and grammar

Chinese characters, vocabulary, and grammar are important components of Chinese language knowledge in relation to CSL acquisition. Chinese is regarded as a difficult language to learn, which is related to both the peculiarities of the Chinese writing system, and the peculiarities of Chinese vocabulary and grammar.

Chinese characters are the written symbols of Chinese and the basis for Chinese reading and writing. CSL learners, especially those from the non-Sinosphere, often find Chinese characters difficult to learn because of fundamental differences between Chinese characters and alphabets regarding stereoscopic structure and ideographic nature (Everson, 1998; Shi and Wan, 1998). First, compared with alphabets’ linear structure, Chinese characters have a square-shaped and more complex structure. Chinese character units comprise strokes and radicals, where strokes are the characters’ smallest units, and radicals are the characters’ secondary units, composed of strokes (Fei, 1996). Although there are few basic strokes in Chinese characters, the strokes have different deformations when in different positions in Chinese characters. For example, the stroke “
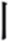
” is “
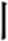
” in the Chinese character “快” (kùai, quick) and is “
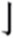
” in the Chinese character “水” (shuiˇ, water). In combining radicals into Chinese characters, the size and direction of strokes in each position also change with the radical’s position and the positional relationship between radicals. For example, the relative position and size of radical “口” in Chinese characters “扣”(kòu, button) and “器”(qì, vessel) differ. Second, Chinese characters belong to the morpheme-syllabic system, with a strong connection between form and meaning, but a poor connection with pronunciation (Tan et al., 2005). Chinese characters comprise four types: pictographic, ideographic, self-explanatory, and pictophonetic, which are all ideographic in nature. For example, the meaning of the pictographic character “刀” (daō, knife) is derived from the graphic “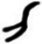,” meaning knife; the meaning of the ideographic character “武”(wuˇ, military) comprises the meaning of two Chinese characters “止”(zhiˇ, stop) and “戈”(gē, dagger-ax), which means the cessation of war; the meaning of the self-explanatory character “刃”(rèn, blade) comprises the meaning of the pictographic character “刀” plus the indicator “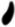,” which means knife blade; the pictophonetic character “湖”(hú, lake) has the semantic radical “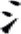,” indicating that the meaning of the character is related to water. Among the four types of Chinese characters, the pictophonetic character is the only one with a phonetic representation function (Li et al., 1992). However, the phonetic radical of the pictophonetic character can no longer accurately represent the pronunciation. For example, “触”(chù, touch) and “浊”(zhuó, muddy), which share the phonetic radical “虫” (chóng, insect), are not pronounced similarly. Chinese characters’ peculiarities make their acquisition tremendously difficult for CSL learners, especially for learners whose L1 is an alphabet script.

Vocabulary is the carrier of meaning, the key to understanding, and the basis of expression. Vocabulary knowledge includes both breadth knowledge and depth knowledge (Meara and Jones, 1988; Zareva et al., 2005; Moinzadeh and Moslehpour, 2012), where breadth knowledge refers to vocabulary size (Qian, 1999), and depth knowledge includes word aggregation relationships (such as synonymous relationships and context relationships) and combinatorial relationships (collocation relationships) (Nation, 1990). From a word aggregation perspective, first, vocabulary learning difficulties are reflected in differences in word concepts’ cognition in different languages. For example, when the Chinese use the word “鱼” (yú, fish) as a metaphor for people, it usually means dishonest, but in Russian, it means silent, and in Czech, it means robust. Second, the degree of word concept refinement differs across languages. Mandarin has a large number of synonyms, many of which have very little difference in meaning. For example: “承继” (chéngjì) and “继承” (jìchéng), both mean subsequent possession, but the former focuses on forward continuation, and the latter on backward continuation. CSL learners often struggle to grasp such subtle differences between words (Zhang, 2019). From a word combination relationships perspective, collocation knowledge reflects syntactic, semantic, and usage frequency information in the mental lexicon (Xing, 2013). Therefore, acquiring collocation knowledge includes mastering both target language collocation grammar rules and collocation words’ semantic category (Shi et al., 2021). However, each language has unique collocation rules, and many collocations are also based on various ethnic groups’ social psychology language habits, which cannot be logically explained (Yamashita and Jiang, 2010). For example, “black tea” in English corresponds to “红茶”(hóngchá) rather than “黑茶”(hēichá) in Chinese. This difference in collocations across languages poses another difficulty in CSL learners’ vocabulary acquisition.

Grammar is the organizational rule of a language; an important sign of second language mastery is mastering its grammar (Li, 2016). Chinese grammar is difficult to learn because, first, unlike Indo-European languages that use a rich variety of morphology to express grammatical relationships, Chinese is an isolated language and lacks strict morphology, so word order and function word are important means of expressing grammatical relationships (Lv, 1979). The grammatical meaning often differs according to the word order. For example, in “我看” (woˇkàn, I see) and “看我” (kànwoˇ, see me), the former indicates a subject-predicate relationship, while the latter indicates a verb-object relationship. The choice of whether to use function words, and the use of different function words, often indicates different grammatical relationships. For example, “买书”(mǎishū) is a verb-object relationship, while “买的书”(mǎideshū) is a subordinate relationship. Second, unlike in English and many other languages, Chinese has no one-to-one correspondence between word class and syntactic constituents (Zhu, 1985)—a word class can act as multiple syntactic constituents, and a syntactic constituent can also be acted on by multiple word classes. Chinese also has some syntactic peculiarities that set it apart from other subject-verb-object languages, such as prepositional phrases followed by verbs, and relative clauses placed before the head. Additionally, Chinese contains unique syntactic constructions, such as pivotal and ba-structure sentences. Therefore, Chinese grammar acquisition difficulties may vary for CSL learners with different L1 backgrounds, owing to these distinctive aspects of Chinese grammar.

However, although Chinese characters, vocabulary, and grammar have distinct characteristics, this does not mean that they are unrelated. From the perspective of Chinese language research, Chinese characters belong to the morpheme-syllabic script, meaning that Chinese characters are not only syllables that represent pronunciation, but also words or morphemes that represent meanings (Li, 2009). The integration of Chinese characters’ form, pronunciation, and meaning has exerted great influence on Chinese words’ syllable form, formation, and meaning composition. Additionally, as Chinese word construction is similar to phrase or sentence construction, syntactic structures and words share a selective restriction relationship (Shi and Yang, 2021); therefore, Chinese character, vocabulary, and grammar characteristics often influence each other. In addition, evidence from many empirical studies shows that knowledge of Chinese characters’ sublexical and grammatical features is also activated during Chinese character processing (Tsai et al., 2004; Yan et al., 2009, 2012; Tsang et al., 2017; Yeh et al., 2017; Pan et al., 2019), which indicates that CSL learners learn Chinese characters, vocabulary, and grammar simultaneously.

In sum, language systems interact across levels, and Chinese character, vocabulary, and grammar characteristics and their acquisition often influence each other. Only when CSL learners’ Chinese characters, vocabulary, and grammar are all well-developed can Chinese proficiency improve. Therefore, these three elements should not be separated when examining CSL learners’ language knowledge acquisition.

### 2.3. Linguistic distance and CSL acquisition

#### 2.3.1. Qualitative linguistic distance and CSL acquisition

Linguistic distance refers to the degree of actual difference between languages and is an important independent variable in the study of cross-language transfer, expressed through intra-linguistic factors, such as phonology, vocabulary, syntax, and writing forms (Ellis, 1994; Chiswick and Miller, 2005). Linguistic distance measures include qualitative and quantitative methods. Studies on CSL acquisition have primarily used qualitative methods, such as genealogical classification and linguistic typological classifications, to examine the influence of linguistic distance on Chinese character, vocabulary, and grammar acquisition.

AS Chinese characters are unique to Chinese, previous studies have compared performance of CSL learners from the Sinosphere and the non-Sinosphere to explore the impact of linguistic distance on Chinese character acquisition. First, linguistic distance has an impact on how quickly CSL learners acquire orthographic awareness of Chinese characters. Several studies have indicated that CSL learners from the Sinosphere develop orthographic awareness more quickly than other CSL learners (Lu, 2002; Feng, 2006; Liu, 2013; Zhang, 2016; Loh et al., 2018). Second, linguistic distance affects CSL learners’ strategies for recognizing Chinese characters. CSL learners from the Sinosphere tend to memorize Chinese characters using their form, and process them using conformational structures; whereas CSL learners from the non-Sinosphere are more likely to be influenced by their L1, relying on phonological strategies to recognize Chinese characters, and processing them using strokes or radicals (Jiang, 2003; Yeh et al., 2003; Feng et al., 2005; Ke and Chan, 2017; Jiang et al., 2020). Third, evidence shows that CSL learners’ L1 orthographic characteristics affect their Chinese character writing, where CSL learners with complex visual space L1 scripts similar to Chinese characters perform better than CSL learners with linear L1 scripts (Lin and Collins, 2012; Zhang and Roberts, 2021). In addition, many studies have compared Chinese character writing and reading performance in CSL learners from different L1 backgrounds and found that Chinese character writing is more difficult than reading. As CSL learners’ Chinese proficiency improves, linguistic distance has fewer effects on Chinese character reading performance, but still has a significant effect on Chinese character writing (Jiang, 2000; Wu et al., 2006; Li et al., 2014; Zhang and Roberts, 2021).

Studies on Chinese vocabulary acquisition level have examined the impact of linguistic distance on the Chinese vocabulary performance of CSL learners from the Sinosphere and non-Sinosphere. Studies on acquiring vocabulary breadth knowledge show that CSL learners’ vocabulary size increases as their Chinese proficiency improves, but at elementary proficiency, CSL learners from the Sinosphere master significantly more vocabulary than CSL learners from the non-Sinosphere (Zhang, 2006; Luo and Duan, 2019). Studies of acquiring vocabulary depth knowledge have generated some controversy regarding vocabulary semantic acquisition. Zhang et al. (2011) compared word semantic acquisition performance of polysemous words between CSL learners from the Sinosphere and the non-Sinosphere at elementary, intermediate, and advanced Chinese proficiency levels and found no significant performance differences in CSL learners from different L1 backgrounds at any Chinese proficiency level. The authors concluded that linguistic distance had no significant effect on CSL learners’ polysemous word acquisition. By contrast, Hong and Chen (2011) found that CSL learners from both the Sinosphere and the non-Sinosphere relied on their L1 to establish L2 synonym semantic relations at elementary Chinese proficiency. Only at intermediate Chinese proficiency could CSL learners significantly acquire the ability to differentiate in lexical semantics. Wang and Hao (2014) reported similar results, finding that CSL learners first acquire knowledge of shared lexical items in both languages at elementary proficiency, and then begin to recognize target language-specific items at intermediate proficiency. The L1-specific items begin to interfere with developing a bilingual mental lexical; only at advanced proficiency do CSL learners gradually abandon the L1-specific items and reach a proficiency close to that of native speakers. Studies on vocabulary collocation knowledge acquisition show relatively consistent results. As Chinese proficiency improves, CSL learners’ vocabulary collocation competence gradually improves (Cai, 2017; Chang, 2020; Shi et al., 2021); even at advanced Chinese proficiency levels, it is influenced by CSL learners’ L1 characteristics, indicating that collocation knowledge with similar L1 and Chinese characteristics is easy to acquire, but that greater difference hinders acquisition (Cai, 2017; Luo and Duan, 2019; Chang, 2020).

Grammar acquisition studies have reported contradictory findings. Some found that similarities between CSL learners’ L1 and Chinese grammatical features promote Chinese grammar acquisition. Guo and Liu (2017) investigated Chinese word order error statistics among CSL learners with intermediate and advanced Chinese proficiency levels, isolated language, agglutinative language, and inflected language L1 backgrounds, and found that the isolated language error rates were lowest with L1 more similar to Chinese, while the inflected language error rate was highest for L1s that differed most from Chinese, indicating that higher similarities between L1 and Chinese facilitate acquisition. Hu et al. (2018) examined the written production of topic-comment constructions by elementary and advanced CSL learners from English and Japanese L1 backgrounds and found that Japanese CSL learners had higher usage rates than English CSL learners at either proficiency level, because Japanese and Chinese are topic-salient languages. Zhang (2021) examined Chinese ellipsis object sentence acquisition by CSL learners with Korean and English L1 backgrounds and also found that CSL learners from English backgrounds with similar characteristics to Chinese showed better acquisition. These findings show that cross-language transfer plays a role in grammar acquisition.

By contrast, some studies found that greater differences between L1 and Chinese better facilitate acquisition. Yuan (2004) examined Chinese negative sentences acquisition in CSL learners from native German, French, and English L1 backgrounds with different learning durations, and found no significant differences in acquisition performance at any proficiency level between CSL learners from English backgrounds whose L1 had similar negation structures to Chinese, or CSL learners from French and German backgrounds whose negation structures were different. The author suggested that this might be because German and French negative structure is quite different from that of Chinese, so it attracts learners’ attention at the beginning, and the difference is constantly strengthened in the process of learning, thus promoting CSL acquisition in learners with German and French backgrounds. Similarly, Wu and Zhao (2018) examined collocation acquisition of Chinese negation markers “不” and “没” with aspect markers “着,” “了,” and “过” by CSL learners from intermediate and advanced English, and Korean backgrounds, and also found that CSL learners from native English backgrounds notice the difference between the collocation of “不” and “没” because English lacks the two negative oppositional markers, thus facilitating acquisition. However, Yuan and Zhao (2005) examined acquisition of resumptive pronouns in Chinese relative clauses by CSL learners with English and Arabic backgrounds, and found that, despite that the use of resumptive pronouns in relative clauses is allowed in Arabic, learners with Arabic backgrounds did not show significantly higher accuracy in judgment tasks than learners with English backgrounds. The authors suggest that learners with Arabic L1 perceive a greater psycho-typological distance between Chinese and Arabic, which hinders positive transfer. Wu and Mo (2018) examined the use of ba-structure sentence among Danish and Korean CSL learners through grammar judgment and picture description tasks and found that, despite that Korean has object prepositions while Danish does not, learners with Danish backgrounds frequently used the ba-structure sentence and were more confident in their understanding of it. According to the retrospective interview, Korean and Chinese object prepositions share some characteristics, but also differ, so learners tended to employ avoidance strategies to lessen usage errors. Furthermore, several studies revealed linguistic distance’s effect on advanced CSL learners’ implicit grammatical processing from an electrophysiological perspective. Hao et al. (2022) used event-related potentials to investigate how linguistic distance affected advanced CSL learners from Indonesia and Thailand acquire Chinese “aspect” and discovered similar EEG patterns evoked by the two learner types for processing aspect violation sentences, but noted that even advanced CSL learners did not reach native speakers’ automatic processing level. These studies hold that psychological typology, learning strategies, and other factors weaken the impact of L1 negative transfer, and they do not entirely deny the role of cross-language transfer.

Linguistic distance’s influence on Chinese character, vocabulary, and grammar acquisition differs by nature and degree. The inconsistent study findings may be related to sampling differences across studies, different L1 backgrounds across studies, and studies being limited to only one language knowledge type of Chinese character, vocabulary, and grammar knowledge. Therefore, analyzing data in relation to CSL learners with larger sample sizes and richer L1 backgrounds is valuable in that it facilitates a fuller understanding of linguistic distance’s influence on CSL knowledge acquisition.

#### 2.3.2. Quantitative linguistic distance and CSL acquisition

Qualitative linguistic distance methods identify differences between languages, but cannot identify the magnitude of the differences (McCloskey, 1998; Chiswick and Miller, 2005); therefore, qualitative methods are significantly limited in comparative studies of learners from several different L1 backgrounds acquiring the same target language. Conversely, quantitative methods determine the numerical magnitude of differences between languages, which facilitates comparing similarities between L1 and target languages and provides a feasible method for calculating linguistic distance. Five quantitative methods for measuring linguistic distance include the dummy variable method, cognate method, test assessment method, automated similarity judgment program (ASJP) edit distance method, and the World Atlas of Language Structures (WALS) index method (Wang and Yang, 2019). The dummy variable method, similar to a qualitative method, dichotomizes linguistic distances and is thus rarely used (Ginsburgh and Weber, 2014). The cognate method calculates the proportion of cognates between languages based on a core word list, but is only applicable to studies between languages within the Indo-European family (Dyen et al., 1992; Schepens et al., 2016). The test assessment method uses language test scores as a linguistic distance measure (Hart-Gonzalez and Lindemann, 1993; Chiswick and Miller, 2005), but is controversial because the results are affected by test reliability, validity, and examinees’ motivation (Van der Slik, 2010). The ASJP edit distance method is based on phonological differences in synonyms or near-synonyms between languages, where fewer conversions indicate a closer linguistic distance (Isphording and Otten, 2013). However, this method calculates the phonological distance between languages, making it more suitable for studies of dialects, languages in which pronunciation features are the main difference, or studies that focus on listening and speaking skills (Isphording and Otten, 2013; Cui et al., 2018). The WALS index method is presently the only method that calculates linguistic distance based on many aspects of language differences, including 192 linguistic features contained in the WALS online^1^ database, which covers 11 categories: phonology, morphology, noun category, noun syntax, verb category, word order, simple sentence, complex sentence, vocabulary, sign language, and others. Therefore, the WALS index method accurately reflects and measures real differences between major languages. It has been used in many studies that compared Chinese with other languages (Bakker et al., 2009; Lupyan and Dale, 2010; Moran and Blasi, 2014; Wang and Cui, 2018; Wang and Yang, 2019; Xu and Zi, 2020).

Studies that quantify linguistic distance to identify second language acquisition influence have primarily been conducted in the language of economics field, with results that reported macro-level effects of L1 to Chinese linguistic distance on language skill acquisition, including listening, speaking, reading, and writing, and consistently reported that shorter linguistic distances between CSL learners’ L1 and Chinese are associated with higher language skill proficiency (Isphording and Otten, 2013; Lindgren and Muñoz, 2013; Schepens et al., 2016; Cui et al., 2018). Language competence includes both language knowledge and language skills, with language knowledge being the basis for language skill development, but evidence suggests that developing language knowledge is not necessarily synchronized with developing language skills. Ding and Xiao (2016) tracked oral expressive skills’ development in Italian CSL learners and found that not all dimensions of vocabulary knowledge were developed as oral proficiency improved. Additionally, Wu (2017) found that as CSL learners’ Chinese proficiency improved, vocabulary and grammar development was not synchronized, and even showed a competitive relationship. Other studies of foreign target language acquisition have reported similar findings (Spoelman and Verspoor, 2010; Lowie et al., 2017). Moreover, the influence of L1 on language knowledge and language skills is not always the same. A meta-analysis (Jeon and Yamashita, 2014) showed that linguistic distance had a significant effect on reading comprehension, but no moderating effect on vocabulary or grammar.

Given this context, it is not possible to simply generalize the findings of studies examining macro-level language skills to the findings of studies examining micro-level language knowledge. Therefore, it is worthwhile to investigate the effect of linguistic distance on CSL acquisition by applying a quantitative method for determining linguistic distance.

## 3. The current study

The literature review showed that a large body of research has examined the influence of linguistic distance on Chinese character, vocabulary, and grammar knowledge acquisition among CSL learners. However, these studies limited the linguistic distance measurement to a comparison of specific features and did not systematically compare differences across languages as a whole. Nor did they systematically investigate linguistic distance’s influence on the acquisition of different language knowledge. Although they applied quantitative methods to calculate linguistic distance and examined the impact of this factor on CSL learners’ acquisition of Chinese based on language tests, they focused only on language skill levels.

Considering the connection between Chinese characters, vocabulary, and grammar, and the complex and dynamic characteristics of language acquisition, the current study used the WALS index method to calculate linguistic distance between CSL learners’ L1 and Chinese, and used HSK test data to systematically investigate the influence of linguistic distance on the acquisition of Chinese characters, vocabulary, and grammar at different proficiency levels. The findings provide systematic empirical support for the role of differences between Chinese and L1 on Chinese language knowledge acquisition.

We conducted three quasi-experiments. Quasi-Experiment 1 examined whether and how linguistic distance affects Chinese character acquisition in CSL learners at elementary, intermediate, and advanced Chinese proficiency levels. Quasi-Experiment 2 examined whether and how linguistic distance affects Chinese vocabulary acquisition in CSL learners at elementary, intermediate, and advanced Chinese proficiency levels. Quasi-Experiment 3 examined whether and how linguistic distance affects Chinese grammar acquisition in CSL learners at elementary, intermediate, and advanced Chinese proficiency levels.

## 4. Quasi-experiment 1

### 4.1. Methods

#### 4.1.1. Participants

Data comprised a subset of a large database (gathered in 2009) that contains information on 80,506 examinees who participated in HSK tests in different regions of China. A brief and non-mandatory questionnaire collected personal background information during online HSK registration, which included basic demographic characteristics, such as gender, age, native language, and place of birth.

We excluded participants with invalid data, including those whose L1 was Chinese or a dialect, or who misfiled or omitted information, resulting in valid data from 58,240 examinees. The examinees spoke 24 L1s (*M* = 2,426.67 speakers per language) that, according to the WALS (Dryer and Haspelmath, 2013), belong to 11 language families (i.e., Afro-Asiatic, Altaic, Austro-Asiatic, Indo-European, Japanese, Korean, Kartvelian, Niger-Congo, Sino-Tibetan, Tai-Kadai, and Uralic).

From each group with elementary, intermediate, and advanced Chinese proficiency, we extracted for inclusion in the analysis 250 CSL learners with a short distance between their L1 and Chinese, 250 with a middle distance, and 250 with a long distance, for a total of 2,250 CSL learners for analysis, owing to the uneven database distribution of CSL learners at different Chinese proficiency levels and different linguistic distance levels (1,121 females; age range 9.70 to 61.80, mean age = 23.44 years, standard deviation = 5.96).

#### 4.1.2. Instruments

The HSK is a national standardized test designed to measure Chinese proficiency in non-native speakers, including international students, overseas Chinese students, and students from ethnic minorities in China. The HSK test score is required for undergraduate or graduate admission to Chinese universities. It also serves as a crucial basis for some business organizations and multinational corporations in China to assess CSL learners’ Chinese communication skills when hiring employees (Meyer, 2014; Teng, 2017).

HSK development has had three stages. HSK Version 1.0 was developed and implemented by Beijing Language and Culture University (BLCU) in 1984. In 2007, BLCU released HSK Version 2.0 to better serve global promotion of the Chinese language. Since 2010, the Confucius Institute Headquarters has improved and perfected the test system and released HSK Version 3.0 (Hanban, 2011).

The study’s HSK data were collected from 78 testing centers in China in 2009. We selected these test data because BLCU has accumulated 26 years of theory and experience on this test (1984–2010), and researchers have conducted sufficient empirical studies on its reliability, construct validity, and test score equivalence to determine that the test results are a reliable indicator of CSL learners’ Chinese proficiency (Chai, 2006; Chen, 2006).

To meet different CSL learner groups’ measurement needs, the designers developed three independent tests: the Elementary HSK, the Intermediate HSK, and the Advanced HSK. The Elementary HSK is only suitable for assessing beginners’ Chinese proficiency; the Advanced HSK assesses CSL learners whose Chinese proficiency is close to that of native Chinese speakers; therefore, the number of examinees for these tests is limited. The Intermediate HSK measures the widest range of Chinese language proficiencies and has the largest number of examinees, so it provides a higher degree of Chinese language proficiency differentiation among CSL learners.

The Intermediate HSK comprises four sections: listening comprehension, grammar structure, reading comprehension, and comprehensive completion, which assess knowledge of Chinese characters, vocabulary, grammar, and listening and reading skills through nine subtests. The Intermediate HSK has a total of 170 points, including 16 for Chinese characters, 20 for vocabulary, and 30 for grammar. Quasi-Experiment 1 focused on the Chinese character subtests.

#### 4.1.3. Variables

In Quasi-Experiment 1, the HSK Chinese character test score was the dependent variable. Two independent between-subjects variables were the linguistic distance between L1 and Chinese, and Chinese proficiency.

##### 4.1.3.1. Chinese character test scores

**Figure 1:**
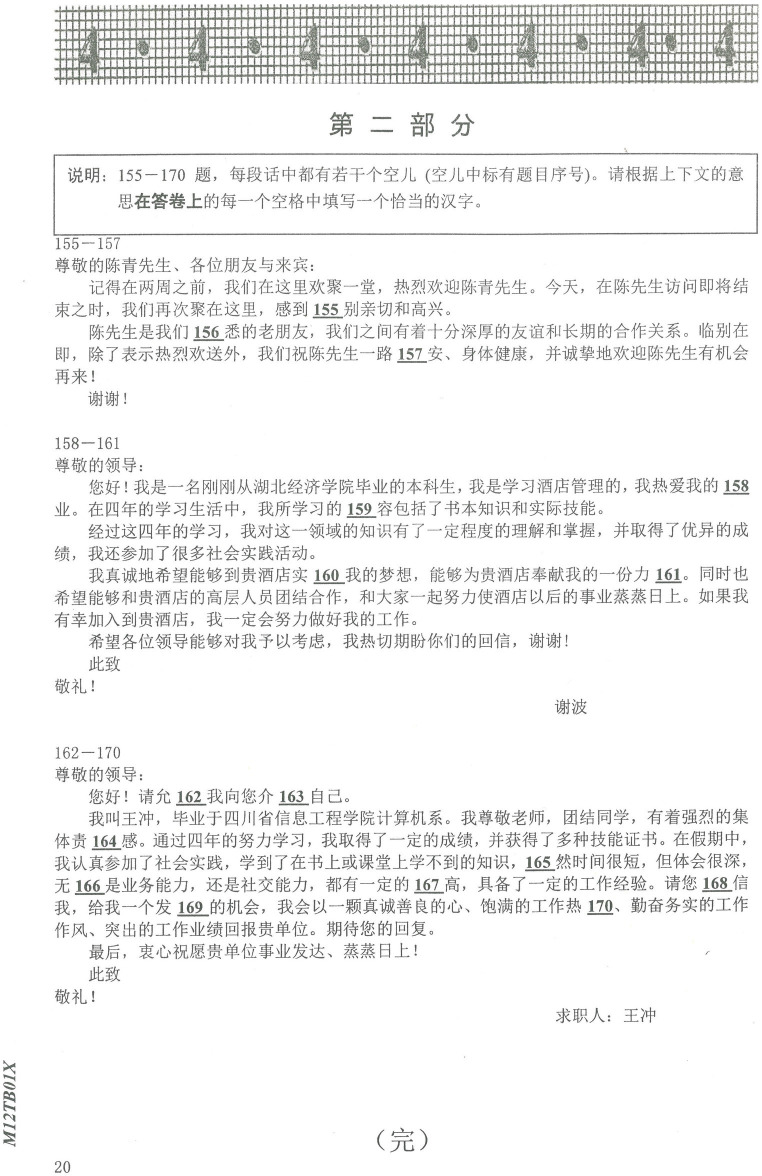
Sample test in the HSK character subtest.

The HSK Chinese character subtest measures orthographic competence by examining CSL learners’ Chinese character writing accuracy under contextual constraints. A higher score on the HSK Chinese character subtest indicates better orthographic proficiency. The Chinese character subtest took 15 min to complete. Figure 1 shows a sample HSK Chinese character subtest.

##### 4.1.3.2. Chinese proficiency

Chinese proficiency was grouped by total HSK score minus the Chinese character test score, to exclude the interference of Chinese character proficiency in the study results. There was a significant positive correlation between total HSK and total HSK minus the Chinese character test scores (*ρ* = 0.995, *p* < 0.001), indicating that excluding the Chinese character scores from the total HSK scores did not change the essential characteristics of the data sample structure.

Therefore, we first ranked the scores from highest to lowest, and then operationally defined the lowest one-third and highest one-third of the examinees as having elementary and advanced Chinese proficiency, respectively, and the middle one-third as having intermediate proficiency. According to this standard, we classified examinees in the full database with scores below 61 as having elementary Chinese proficiency (*n* = 9,493); those with scores between 62 and 107 as having intermediate Chinese proficiency (*n* = 33,093); and those with scores above 108 as having advanced Chinese proficiency (*n* = 15,654).

##### 4.1.3.3. Linguistic distance

We computed linguistic distance between participants’ L1 and Chinese based on the linguistic structural features contained in the online WALS. First, based on 153 Chinese features, we compared feature similarities and differences between CSL learners’ L1 and Chinese. Common features of both languages were assigned 0, and different features were assigned 1. We then calculated the average value of the assignment results of each L1, with lower linguistic similarity between L1 and Chinese indicating a higher WALS value. The linguistic distance values ranged from 0.33 to 0.64 (*M =* 0.49, *SD =* 0.08). Based on the linguistic distance value ranges, we divided the 24 L1s into three equal groups; the short-distance group included participants with linguistic distance values below 0.44, the middle-distance group had values between 0.44 and 0.54, and the long-distance group had distance values above 0.54. Table 1 shows the descriptive statistics for each linguistic distance level group in the overall database sample.

**Table 1:** Participant descriptive statistics with the linguistic distance between L1s and Chinese.

**Linguistic distance levels**	**L1**	**Values**	***N***
Short	Vietnamese	0.33	3,888
	Tai	0.34	3,468
	Korean	0.35	29,012
	Indonesian	0.40	1,837
	Yoruba	0.42	2
	Japanese	0.43	10,966
Middle	Burmese	0.45	159
	English	0.47	1,889
	Hausa	0.49	2
	Fijian	0.49	3
	Tagalog	0.50	42
	Russian	0.50	3,742
	Finnish	0.51	43
	Spanish	0.52	515
	Hungarian	0.52	34
	Hindi	0.53	230
	Hebrew	0.53	34
	Turkish	0.53	486
Long	Greek	0.56	16
	Persian	0.56	37
	German	0.57	668
	Swahili	0.60	33
	French	0.62	1,123
	Georgian	0.64	11

#### 4.1.4. Data analysis

We used a two-way between-subjects ANOVA to test for a main effect of linguistic distance on Chinese character acquisition and an interaction effect between linguistic distance and Chinese proficiency. It should be noted that several authors have argued that violation of normality is not a serious problem (Sokal and Rohlf, 1995, p. 407; Zar, 2010, p. 137) in terms of the central limit theory. Some authors even argue that the normality assumption is not needed with adequately large samples (Fitzmaurice et al., 2012; Ghasemi and Zahediasl, 2012). Therefore, we used two-way between-subjects ANOVA for data analysis, even if the data did not meet the normality assumption.

We used the Sidak method to correct for significance levels when performing *post hoc* multiple comparisons of linguistic distance main effects, and simple effects tests for the interaction between linguistic distance and Chinese proficiency. We calculated effect sizes using partial eta squared (*η*2*p*) and classified effect sizes as very small (0–0.02); small (0.02–0.15); moderate (0.15–0.35); and large (0.35–1.0; Cohen, 1992). We considered two-tailed probability values <0.05 statistically significant.

All analyzes were performed using IBM SPSS Statistics version 26.0; data visualization was performed using the R statistical programming language.

### 4.2. Results

To examine whether and how linguistic distance affects Chinese character acquisition by elementary, intermediate, and advanced proficiency CSL learners, we analyzed Chinese character test scores from 2,250 CSL learners in nine groups. Table 2 shows the Chinese character test score descriptive statistics for the nine CSL learner groups.

**Table 2:** Chinese character test score descriptive statistics.

**Chinese proficiency level**	**Linguistic distance level**	**Mean values of Chinese characters**
Elementary	Short	3.52 (SD = 2.19)
	Middle	2.83 (SD = 2.11)
	Long	2.80 (SD = 1.80)
Intermediate	Short	7.37 (SD = 3.07)
	Middle	6.01 (SD = 3.11)
	Long	5.58 (SD = 2.80)
Advanced	Short	10.93 (SD = 2.83)
	Middle	9.50 (SD = 3.74)
	Long	9.29 (SD = 3.26)

**Figure 2:**
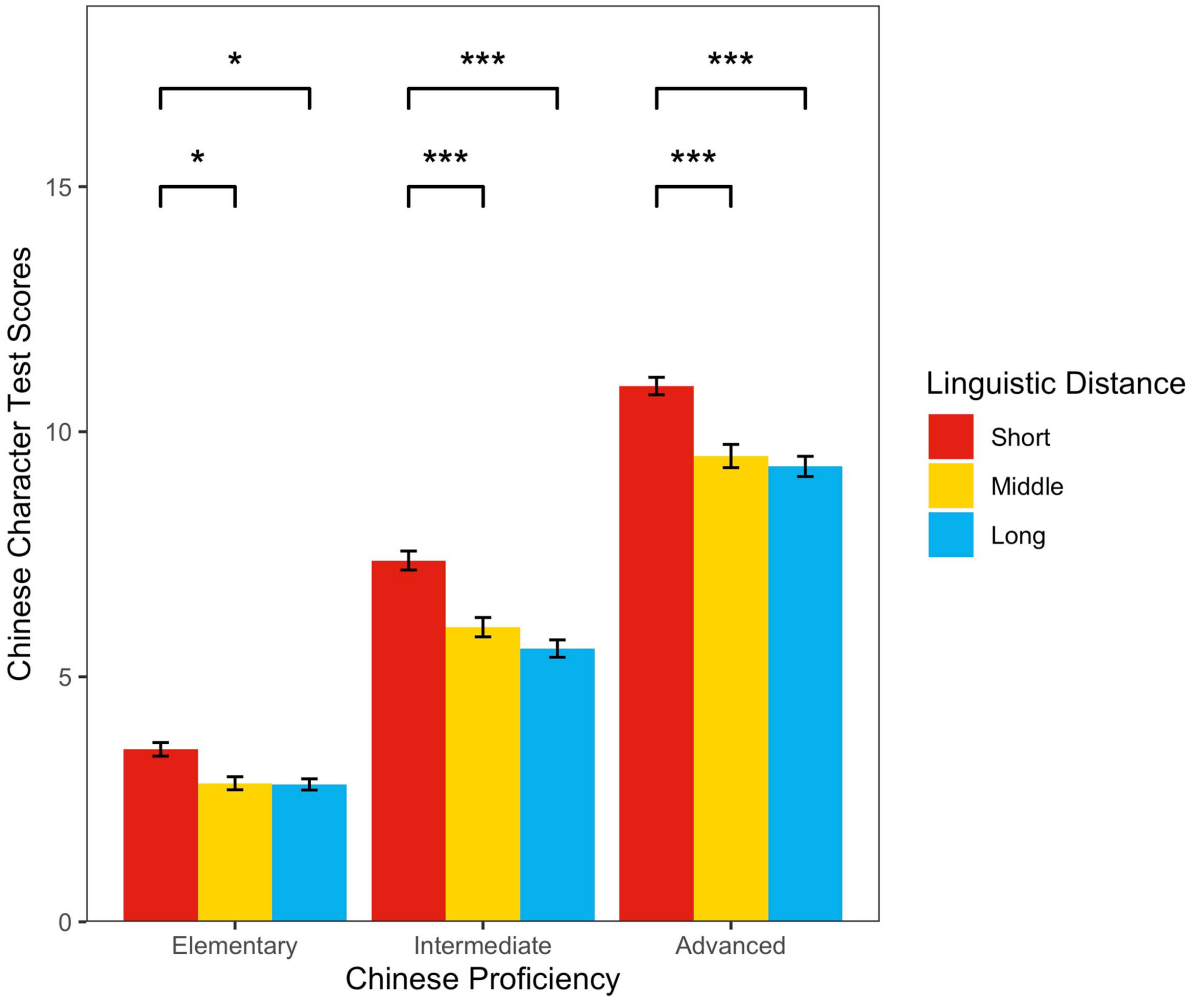
The relationship between Chinese proficiency, linguistic distance, and Chinese character test scores.

Two-way between-subjects ANOVAs revealed a significant main effect of linguistic distance (*F*_(2，2,241)_ = 51.69, *p* < 0.001, *η_p_^2^* = 0.044) and Chinese proficiency (*F*_(2，2,241)_ = 1102.23, *p* < 0.001, *η_p_^2^*_=_0.496), with a significant interaction effect (*F*_(4，2,241)_ = 2.86, *p* = 0.02, *η_p_^2^* = 0.005). To further explore specific differences between the groups, we conducted a simple effects test, which showed that at the elementary Chinese proficiency level, the short-distance group Chinese character scores were significantly higher than those of the middle-distance group (mean difference = 0.69, *p* = 0.02) and long-distance group (mean difference = 0.72, *p* = 0.01). Although the middle-distance group’s Chinese character scores were higher than those of the long-distance group, the difference was not significant (mean difference = 0.024, *p* = 1.00). At the intermediate Chinese proficiency level, the short-distance group’s Chinese character scores were significantly higher than those of the middle-distance group (mean difference = 1.36, *p* < 0.001); the short-distance group scores were also significantly higher than those of the long-distance group (mean difference = 1.80, *p* < 0.001). Although the middle-distance group’s performance was higher than that of the long-distance group, the difference was not significant (mean difference = 0.44, *p* = 0.23). At the advanced Chinese proficiency level, the short-distance group’s Chinese character scores were significantly higher than those in the middle-distance group (mean difference = 1.43, *p* < 0.001); the short-distance group’s Chinese character scores were also significantly higher than those in the long-distance group (mean difference = 1.64, *p* < 0.001), but there was no significant difference between the middle-distance group and the long-distance group (mean difference = 0.21, *p* = 0.79; see Figure 2).

The findings from Quasi-Experiment 1 showed how linguistic distance affected CSL learners’ Chinese character acquisition, where performance differed across the elementary, intermediate, and advanced Chinese proficiency conditions, but the short-distance group’s Chinese character performance was noticeably better than that of the middle-and long-distance groups. Therefore, differences in linguistic distance’s influence on CSL learners’ Chinese character acquisition predominately manifested between the short-and middle-distance groups, and between the short-and long-distance groups.

## 5. Quasi-experiment 2

### 5.1. Methods

#### 5.1.1. Participants

Quasi-Experiment 2 data came from the same database as Quasi-Experiment 1. For comparable results between the two experiments, we extracted 250 CSL learners with a short distance between L1 and Chinese, 250 with a middle distance, and 250 with a long distance from each of the elementary, intermediate, and advanced Chinese proficiency groups, for a total of 2,250 individual HSK vocabulary scores for analysis (1,161 females; age range 9.90 to 67.4 years, mean age = 23.33, standard deviation = 5.85).

#### 5.1.2. Variables

The Quasi-Experiment 2 dependent variable was HSK vocabulary test scores. The two independent between-subject variables were the linguistic distance between L1 and Chinese, and Chinese proficiency.

##### 5.1.2.1. Vocabulary test scores

**Figure 3:**
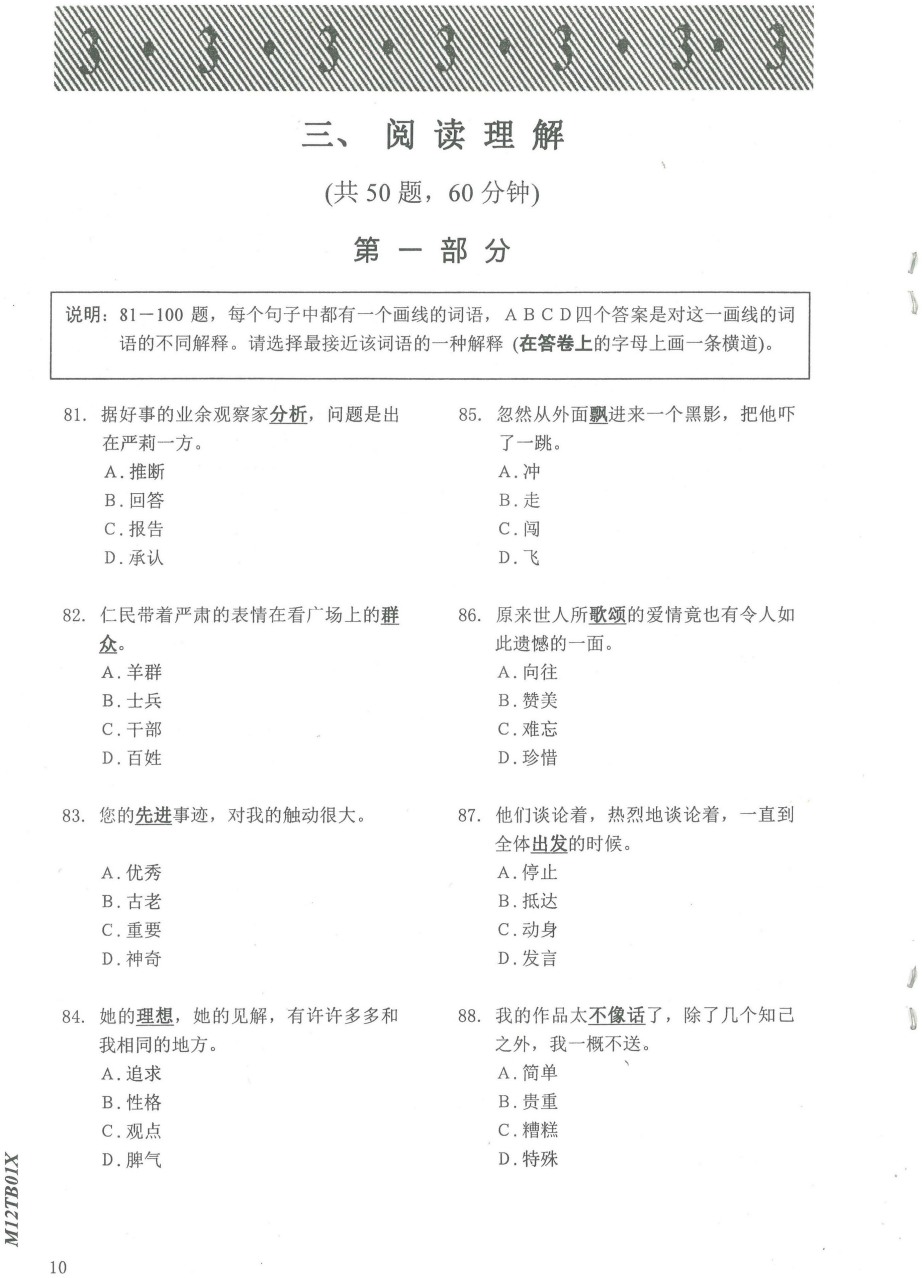
Sample test in the HSK vocabulary subtest.

Vocabulary scores were derived from the HSK vocabulary subtests, which measure vocabulary knowledge depth and breadth, with higher HSK vocabulary scores indicating higher vocabulary proficiency. The vocabulary subtest took 20 min to complete. A sample of the test is shown in Figure 3.

##### 5.1.2.2. Chinese proficiency

Similar to the approach used in Quasi-Experiment 1, Quasi-Experiment 2 divided Chinese proficiency into three groups according to the total HSK scores minus the vocabulary scores. We found a significant positive correlation between total HSK score and total HSK score minus the vocabulary score (*ρ* = 0.996, *p* < 0.001). Finally, in the full database, we classified scores below 61 as elementary Chinese proficiency (*n* = 9,404); scores between 62 and 107 as intermediate Chinese proficiency (*n* = 34,041); and scores above 108 as advanced Chinese proficiency (*n* = 14,795).

##### 5.1.2.3. Linguistic distance

The calculation and grouping methods for linguistic distance were the same as in Quasi-Experiment 1.

#### 5.1.3. Data analysis

Data analysis methods and procedures were the same as in Quasi-Experiment 1.

### 5.2. Results

To examine whether and how linguistic distance affects Chinese vocabulary acquisition by elementary, intermediate, and advanced proficiency CSL learners, we analyzed HSK vocabulary test scores from 2,250 CSL learners in nine groups. Table 3 shows Chinese vocabulary test score descriptive statistics for the nine CSL learner groups.

**Table 3:** Chinese vocabulary test score descriptive statistics.

**Chinese proficiency level**	**Linguistic distance level**	**Mean values of Chinese vocabulary**
Elementary	Short	7.44 (SD = 2.80)
	Middle	6.31 (SD = 2.42)
	Long	6.06 (SD = 2.57)
Intermediate	Short	11.91 (SD = 3.13)
	Middle	10.86 (SD = 3.42)
	Long	10.29 (SD = 3.12)
Advanced	Short	16.33 (SD = 2.22)
	Middle	16.22 (SD = 2.50)
	Long	15.34 (SD = 2.57)

Two-way between-subjects ANOVAs revealed a significant main effect of linguistic distance (*F*_(2，2,241)_ = 43.41, *p* < 0.001, *η_p_^2^* = 0.037) and Chinese proficiency (*F*_(2，2,241)_ = 2135.19, *p* < 0.001, *η_p_^2^* = 0.656), with a significant interaction effect (*F*_(4，2,241)_ = 2.86, *p* = 0.02, *η_p_^2^* = 0.005).

**Figure 4:**
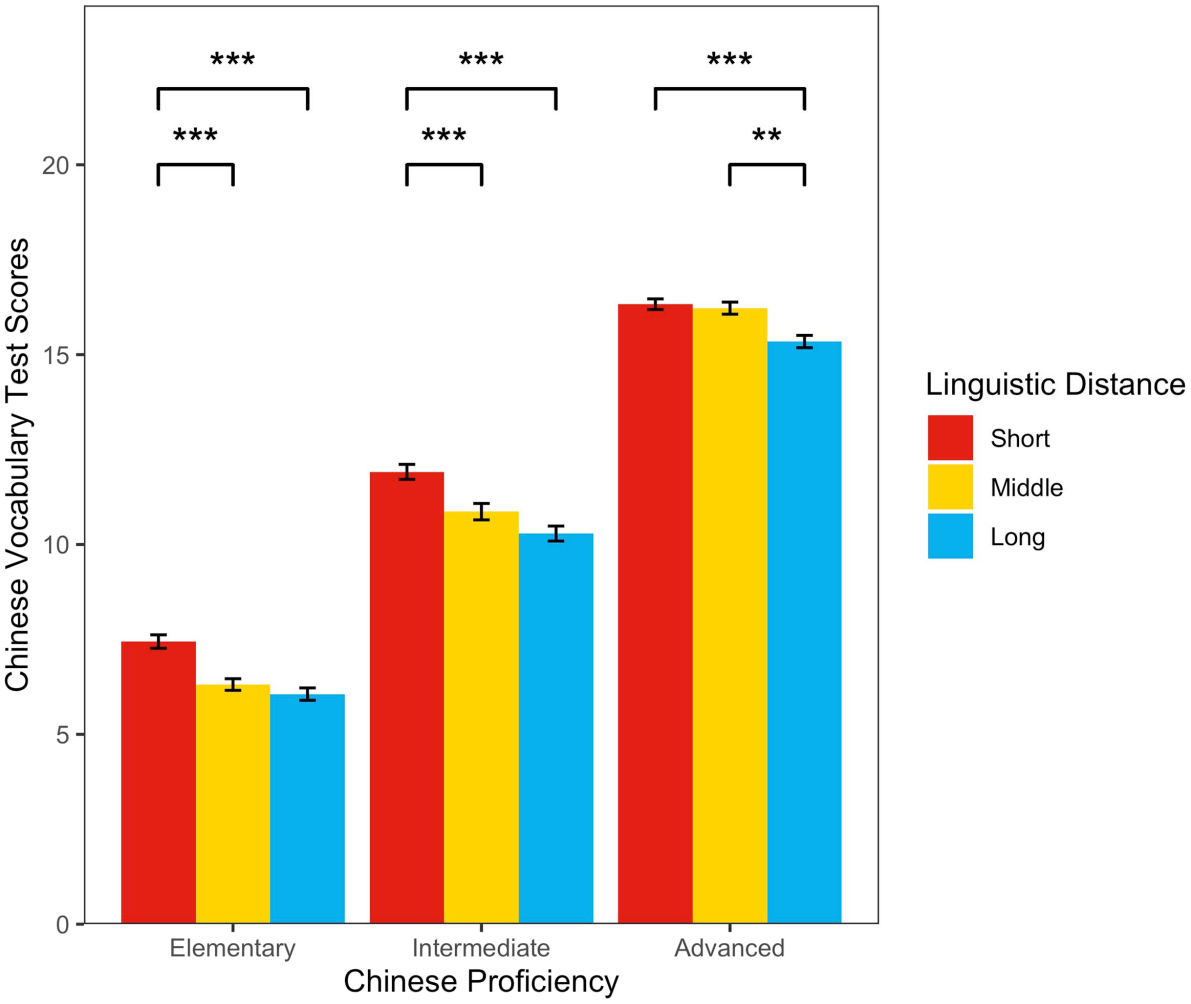
The relationship between Chinese proficiency, linguistic distance, and Chinese vocabulary test scores.

A simple effect test showed that for elementary Chinese proficiency, the short-distance group’s vocabulary scores were significantly higher than those of the middle-distance group (mean difference = 1.13, *p* < 0.001), and those of the long-distance group (mean difference = 1.38, *p* < 0.001). However, there was no significant difference between the middle-distance and long-distance groups (mean difference = 0.25, *p* = 0.67). For intermediate Chinese proficiency, the short-distance group’s vocabulary scores were significantly higher than those of the middle-distance (mean difference = 1.05, *p* < 0.001), and long-distanc (mean difference = 1.62, *p* < 0.001) groups. However, there was no significant difference between the middle-distance and long-distance groups’ scores (mean difference = 0.58, *p* = 0.06). For advanced Chinese proficiency, there was no significant difference between the short-distance and middle-distance groups’ vocabulary scores (mean difference = 0.10, *p* = 0.97), but the two groups’ scores were significantly higher than those of the long-distance group (mean difference = 0.98, *p* < 0.001, and mean difference = 0.88, *p* = 0.001, respectively) (see Figure 4).

Quasi-Experiment 2 showed that linguistic distance affected CSL learners’ vocabulary acquisition, and that vocabulary performance differed across distance groups under the elementary, intermediate, and advanced Chinese proficiency conditions. First, the short-distance group’s vocabulary scores were significantly higher than those of the middle-distance group in the elementary and intermediate Chinese proficiency conditions, but there was no significant difference between the two groups in the advanced Chinese proficiency condition. This indicates that difference in vocabulary acquisition performance between the short-and middle-distance groups decreased as Chinese proficiency increased. Second, there was no significant difference between the middle-distance and long-distance groups in the elementary and intermediate proficiency conditions, but the middle-distance group had significantly higher vocabulary scores than the long-distance group under the advanced Chinese proficiency condition. This indicates that as Chinese proficiency improved, vocabulary acquisition performance differences between the middle-distance and the long-distance groups increased. Finally, although the difference in vocabulary scores between the short-and long-distance groups showed a decreasing trend, the short-distance group’s vocabulary scores were significantly higher than those of the long-distance group at any Chinese proficiency level.

## 6. Quasi-experiment 3

### 6.1. Methods

#### 6.1.1. Participants

Quasi-Experiment 3 data came from the same database as the previous two quasi-experiments, and the method and number of participants were also selected in the same way as for Quasi-Experiments 1 and 2 (1,136 females; age range 11.2 to 61.9 years, mean age = 23.53, standard deviation = 5.91).

#### 6.1.2. Variables

The Quasi-Experiment 3 dependent variable was HSK grammar scores, and the two independent between-subject variables were linguistic distance between L1 and Chinese, and Chinese proficiency.

##### 6.1.2.1. Grammar test scores

**Figure 5:**
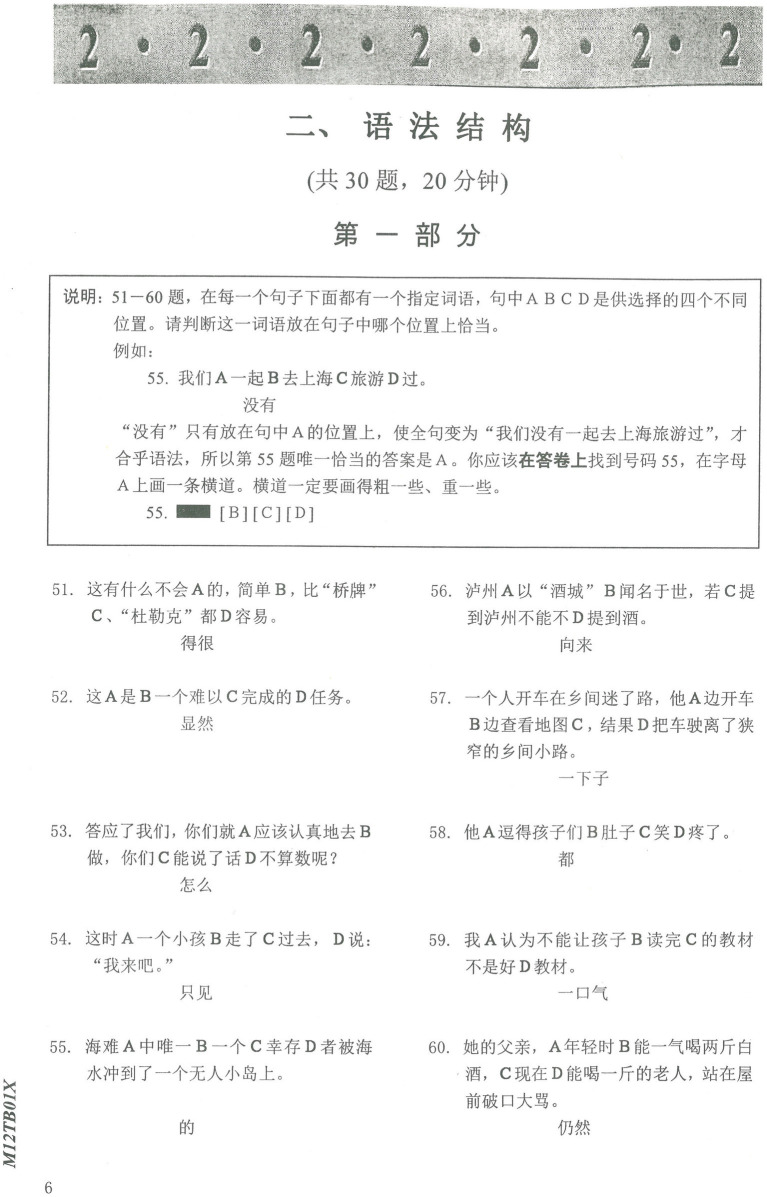
Sample test in the HSK vocabulary subtest.

Grammar scores were derived from the HSK grammar subtests, which measure Chinese grammar knowledge by examining grammar usage accuracy, where higher grammar subtest scores indicate higher grammar proficiency. The grammar subtest takes 30 min to complete. Figure 5 shows a grammar subtest sample.

##### 6.1.2.2. Chinese proficiency

Quasi-Experiment 3 grouped Chinese proficiency the same as in quasi-experiments 1 and 2. There was a significant positive correlation between total HSK score and total HSK score minus the grammar subtest score (*ρ =* 0.993, *p <* 0.001). Scores less than 56 were defined as elementary proficiency (*n* = 10,510); scores between 57 and 98 as intermediate proficiency (*n* = 33,305); and scores above 99 as advanced proficiency (*n* = 14,425).

##### 6.1.2.3. Linguistic distance

The linguistic distance calculation and grouping methods were the same as in Quasi-Experiments 1 and 2.

#### 6.1.3. Data analysis

The data analysis approach and process were the same as for Quasi-Experiments 1 and 2.

### 6.2. Results

To examine whether and how linguistic distance affected Chinese grammar acquisition by elementary, intermediate, and advanced proficiency, we analyzed the HSK grammar scores of 2,250 CSL learners in nine groups. Table 4 shows the grammar test score descriptive statistics for the nine groups.

**Table 4:** Chinese grammar test score descriptive statistics.

**Chinese proficiency level**	**Linguistic distance level**	**Mean values of Chinese grammar**
Elementary	Short	10.68 (SD = 3.14)
	Middle	10.83 (SD = 3.86)
	Long	11.26 (SD = 3.67)
Intermediate	Short	16.96 (SD = 4.19)
	Middle	17.82 (SD = 4.41)
	Long	17.99 (SD = 4.25)
Advanced	Short	23.52 (SD = 3.63)
	Middle	25.36 (SD = 3.18)
	Long	24.75 (SD = 2.96)

Two-way between-subjects ANOVA revealed a significant main effect of linguistic distance (*F*_(2，2,241)_ = 16.111, *p* < 0.001, *η_p_^2^* = 0.014) and Chinese proficiency (*F*_(2，2,241)_ = 2499.552, *p* < 0.001, *η_p_^2^* = 0.690), and a significant interaction effect (*F*_(4，2,241)_ = 3.323, *p* = 0.01, *η_p_^2^* = 0.006).

**Figure 6:**
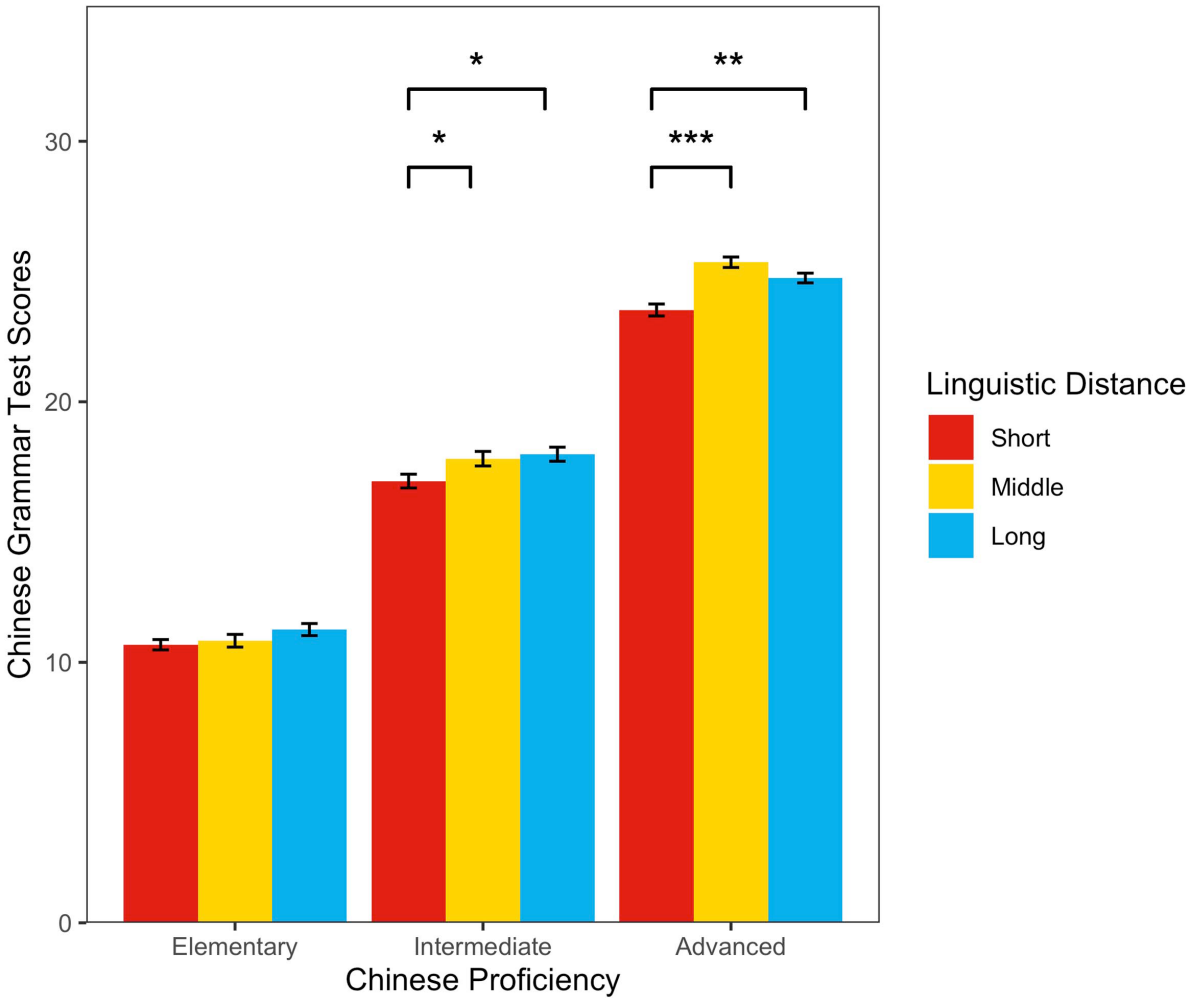
The relationship between Chinese proficiency, linguistic distance, and Chinese grammar test scores.

The simple effect test showed that the elementary proficiency group had no significant difference in grammar scores across the long-, middle-, and short-distance groups (mean difference = 0.58, *p =* 0.23; mean difference = 0.43, *p =* 0.49; mean difference = 0.15, *p =* 0.96, respectively). The intermediate proficiency group’s long-distance grammar scores were significantly higher than those of the short-distance group (mean difference = 1.03, *p* = 0.01). The middle-distance group’s grammar scores were also significantly higher than those of the short-distance group (mean difference = 0.86, *p* = 0.03), but there was no significant difference between the long-and middle-distance groups (mean difference = 0.17, *p* = 0.94). For advanced proficiency, the long-distance group’s grammar scores were significantly higher than those of the short-distance group (mean difference = 1.23, *p* = 0.001); the middle-distance group’s grammar scores were also significantly higher than those of the short-distance group (mean difference = 1.83, *p* < 0.001). Although the middle-distance group’s average score was higher than that of the long-distance group, the difference was not significant (mean difference = 0.60, *p* = 0.20) (see Figure 6).

Quasi-Experiment 3 showed that linguistic distance affected CSL learners’ grammar acquisition, and that distance groups’ grammar scores were not consistent across the elementary, intermediate, and advanced proficiency groups. Under elementary Chinese proficiency, there was no significant difference in grammar scores across the distance groups. However, under intermediate and advanced proficiency, the middle-and long-distance groups’ grammar scores were significantly higher than those of the short-distance group, indicating that CSL grammar acquisition differences increased as Chinese proficiency improved, as shown between the long-and short-distance groups, and the middle-and short-distance groups.

## 7. General discussion

This study explored the effects of linguistic distance on CSL learners’ Chinese character, vocabulary, and grammar knowledge acquisition by Chinese proficiency level. The results fully demonstrated the complexity and dynamics of L1 difference effects in language knowledge acquisition. First, the Chinese character acquisition results showed that shorter linguistic distance between L1 and Chinese was associated with better acquisition across the elementary, intermediate, and advanced Chinese proficiency levels. Second, the vocabulary acquisition results showed that, at elementary, intermediate, and advanced proficiency levels, shorter linguistic distance between L1 and Chinese was associated with better acquisition. However, the difference in vocabulary acquisition performance between the short-and middle-distance groups gradually decreased as learners’ Chinese proficiency improved, while the difference in vocabulary acquisition performance between the long-and middle-distance groups gradually increased. Third, the grammar acquisition results showed that linguistic distance did not significantly affect CSL learners’ grammar acquisition for those with elementary Chinese proficiency, but at intermediate and advanced Chinese proficiency, longer linguistic distance was beneficial to grammar acquisition. These results indicate that only Chinese character and vocabulary acquisition support the cross-language transfer theory hypothesis; grammar acquisition does not support the theory.

### 7.1. Linguistic distance and Chinese character acquisition

Quasi-Experiment 1 examined linguistic distance effects on CSL learners’ Chinese character acquisition at different Chinese proficiency levels. The results showed that, for elementary, intermediate, or advanced Chinese proficiency levels, Chinese character acquisition was more favorable with a shorter linguistic distance between L1 and Chinese, but this effect was only observed between the short-and middle-distance groups and the short-and long-distance groups. There were six L1s in the short-distance group comprising Vietnamese, Thai, Korean, Indonesian, Yoruba, and Japanese individuals. Japan, Korea, and Vietnam are Sinosphere member countries, while Indonesia and Thailand are both neighbors of China. Thus, our findings are consistent with the findings of most previous studies that Chinese characters are better acquired in Sinosphere CSL learners than in non-Sinosphere CSL learners (Jiang, 2003; Feng et al., 2005; Zhang, 2017; Loh et al., 2018; Deng and Hu, 2022). This result is partially consistent with cross-language transfer theory assumptions. According to previous studies, CSL learners in short-distance groups (mainly from the Sinosphere) have a basic understanding of the rules for writing Chinese characters because their native script has Chinese character forms, and they can acquire Chinese character meanings much easier because they have more frequent exposure to Chinese culture. Therefore, short-distance CSL learners have an advantage in both Chinese character experience and literacy (Feng et al., 2005; Feng, 2006; Lin and Collins, 2012). By contrast, the middle-and long-distance CSL learners have alphabetic language backgrounds; the stereoscopic structure of Chinese characters is more complex compared to the linear structure of alphabetic characters (Everson, 1998; Shi and Wan, 1998). Additionally, literary strategies in relation to Chinese characters differ greatly from those of alphabetic characters (Tan et al., 2005). The form-phonemic connection of Chinese characters is poor; however, CSL learners from alphabetic backgrounds are often influenced by their L1 and tend to use native-like form-phonemic strategies to recognize Chinese characters (Jiang, 2003). Moreover, there is a long-standing lack of emphasis on Chinese characters when teaching CSL (Li, 2017). All these factors increase the difficulty of Chinese character acquisition for CSL learners from non-Sinosphere countries, resulting in their inability to avoid the influence of negative cross-language transfer on Chinese character acquisition, even if they reach a relatively advanced Chinese proficiency level.

### 7.2. Linguistic distance and Chinese vocabulary acquisition

Quasi-Experiment 2 examined the influence of linguistic distance on CSL learners’ vocabulary acquisition at different proficiency levels. The results showed that CSL learners with elementary and intermediate proficiency and short linguistic distance had a vocabulary acquisition advantage, and that CSL learners with advanced proficiency and either middle or short linguistic distance showed significantly better performance than those with a long linguistic difference. First, these results are partially consistent with the finding that shorter linguistic distance is more favorable to vocabulary acquisition, which accords with cross-language transfer theory and most previous study findings (Zhang, 2006; Cai, 2017; Luo and Duan, 2019; Chang, 2020). Previous studies have shown that, because Sinosphere L1s share many Chinese origin words and morphemes, CSL learners from Sinosphere countries have a certain awareness of Chinese morphemes and can use morphemic strategies to learn new words at the elementary stage (Hong, 2011; Xu, 2014). Shorter linguistic distance to Chinese often means more exposure to Chinese culture; since words in different languages can be linked through concepts (Zhang et al., 2011), shorter linguistic distance also means easier conceptual word linkage, which makes positive cross-language transfer easier.

Second, vocabulary scores of all distance groups significantly increased with improvements in CSL learners’ Chinese proficiency levels. Vocabulary score differences between the short-and middle-distance groups significantly decreased, but vocabulary score differences between the long-and middle-distance groups significantly increased. We believe this result reflects a difference in the linguistic distance effect on vocabulary acquisition speed for non-Sinosphere CSL learners. As the middle-distance group had a linguistic distance advantage over the long-distance group, vocabulary knowledge expansion with improved Chinese proficiency could reduce the effect of negative cross-language transfer; whereas CSL learners in the long-distance group, whose L1 differed more from Chinese, were more affected by negative cross-language transfer, requiring them to overcome more vocabulary acquisition difficulties, which resulted in significantly slower vocabulary acquisition speeds for CSL learners in the long-distance group compared to the middle-distance group. Combined with the trend (although not significant) for decreased vocabulary score differences between the short-and long-distance groups, we believe that linguistic distance’s influence on CSL learners’ vocabulary acquisition generally diminished with increased Chinese proficiency.

Additionally, in comparing the results with those of Quasi-Experiment 1, we found similarities in the effects of linguistic distance on Chinese character and vocabulary acquisition, where shorter linguistic distance between L1 and Chinese was associated with more favorable acquisition, and showed a significant acquisition advantage for Sinosphere CSL learners at any proficiency level. This is most likely a result of Chinese characters and words being similarly acquired because of their blurred boundaries (Li, 2009). However, there were also differences in linguistic distance effects on character and vocabulary acquisition. Quasi-Experiment 2 showed no significant difference in vocabulary performance between the short-and middle-distance groups for CSL learners with advanced Chinese proficiency, while Quasi-Experiment 1 showed a significant difference. Considering that the HSK Chinese character subtest examines learners’ Chinese character or morpheme writing, and the vocabulary subtest focuses on word or morpheme recognition, this result supports, to some extent, the finding that Chinese character or morpheme writing is more influenced by linguistic distance than is reading (Wu et al., 2006; Liu, 2008; Li et al., 2014). Based on previous studies, we believe that the main reason for this phenomenon is that Chinese character recognition can be accomplished by using only part of a character’s information to remember its pronunciation or meaning, so it is only necessary to break these characters down into strokes; whereas writing requires learners to recall and reproduce the characters through their pronunciation and meaning, which requires mastery of not only the character radicals, but also the smaller stroke units (Jiang, 2000; Yeh et al., 2003; Xu Y. et al., 2013; Ke and Chan, 2017). Therefore, writing Chinese characters or morphemes is more difficult, resulting in a more profound negative cross-language transfer effect on Chinese character writing.

### 7.3. Linguistic distance and Chinese grammar acquisition

Interestingly, Quasi-Experiment 3 showed a pattern of cross-language transfer effects that completely differed from those found in Chinese character and vocabulary acquisition. There was no significant linguistic distance effect on grammar acquisition for CSL learners with elementary Chinese proficiency. This may be because cross-language transfer is evident in early stages, but in the middle-and long-distance groups, linguistic differences’ promoting effect weakened the L1 negative transfer effects and facilitated acquisition (Yuan, 2004; Wu and Zhao, 2018). Meanwhile, numerous studies have demonstrated that elementary proficiency learners rely more on lexical semantic information than syntactic information (Clahsen and Felser, 2006, 2018; Rah and Adone, 2010); therefore, grammatical knowledge development frequently lags behind that of content meaning knowledge, such as Chinese characters and vocabulary, which may be another reason why L1 differences are unlikely to show a significant grammar acquisition effect for CSL learners with elementary Chinese proficiency.

Although linguistic distance influenced CSL learners’ grammar acquisition at intermediate and advanced proficiency levels, short distance did not contribute to grammar acquisition at these proficiency levels. In other words, short distance did not produce a positive cross-language transfer, but rather had a hindering effect.

Previous studies suggest that this result may stem from a greater difference between learners’ L1 and Chinese, which makes learners more likely to pay attention to the differences, facilitating acquisition (Yuan, 2004; Wu and Zhao, 2018); or it could be that a high degree of cross-language similarity makes learners more likely to ignore differences, making acquisition more difficult (Laufer, 1990; Ellis, 1999). This result can only occur when language features’ similarity interference effects are significantly stronger than language differences’ facilitation effects, and significantly stronger than L1 transfer effects. Additionally, with increased grammar learning content, the probability of overgeneralizing will be greater (Ellis, 1994); compared with obvious differences between learners’ L1 and target language, the probability of overgeneralizing subtle differences between learners’ L1 and target language is greater (Ellis, 1999). In other words, as Chinese proficiency improves and CSL learners are exposed to more grammatical knowledge, short-distance group CSL learners are not only subject to interference from certain grammar with highly similar features to Chinese, but also to interference from their native grammar. This may result in a longer period of confusion before learners fully master Chinese grammar. Therefore, it is reasonable to believe that enhanced interference effects of short-distance L1 on CSL learners’ grammar acquisition inhibits grammatical competence development as their Chinese proficiency improves.

Previous studies showed that avoidance strategies and psycho-typological distance weaken L1 negative transfer and result in a non-significant acquisition advantage for short-distance CSL learners. However, as the HSK is a high-risk test with objective items, examinees are less likely to use avoidance strategies. The role of learners’ perceived differences in language typology is beyond the scope of our study; these issues can be further explored in future studies.

Our study also found no significant differences in Chinese character, vocabulary, and grammar scores between different distance groups at each Chinese proficiency level, except for vocabulary scores for CSL learners with advanced Chinese proficiency. This finding indicates that linguistic distance is important for distinguishing language knowledge acquisition performance in both Sinosphere and non-Sinosphere CSL learners, but does not play an obvious role in distinguishing language knowledge acquisition performance within non-Sinosphere CSL learners. Given the lack of studies that specifically focus on differences in Chinese acquisition within non-Sinosphere CSL learners, we cannot yet offer a general explanation.

Our results show that linguistic distance has different patterns of influence on Chinese character, vocabulary, and grammar acquisition. As Ellis (1999) asserted, L1’s influence on second language acquisition is not always a one-way positive or negative process, but involves a reciprocally dynamic process (Xu Q. et al., 2013).

### 7.4. Differences in the influence of linguistic distance on language knowledge and language skills

Previous studies showed that shorter linguistic distances between L1 and Chinese facilitate CSL learners’ language skill development, such as listening, speaking, reading, and writing (Wang and Cui, 2018; Wang and Yang, 2019; Xu and Zi, 2020). Using HSK test data, we investigated the impact of linguistic distance between L1 and Chinese on character, vocabulary, and grammar knowledge acquisition. The results showed differences in linguistic distance’s effect on the total characteristics of three types of language knowledge, and differences in the degree of influence on three types of language knowledge acquisition at different Chinese language proficiency levels. Our findings indicate that, at least under the influence of linguistic distance, language knowledge acquisition is not consistent with language skills acquisition, because the language knowledge acquisition process is more complex (Ding and Xiao, 2016; Wu, 2017).

## 8. Limitations and future directions

The current study has limitations. First, the linguistic features provided by the WALS online system for calculating cross-language similarity between L1 and Chinese vary in both the number of indicators and their categories. We could only examine test data from 24 L1 backgrounds that do not differ significantly from the number of Chinese linguistic features.

Second, the study only examined acquisition performance of intermediate HSK examinees at different proficiency levels, so the results do not reflect higher proficiency learners’ acquisition characteristics. Future research using larger samples with richer L1 backgrounds is recommended.

Third, internal factors have important effects on second language acquisition, such as learners’ L1, language proficiency and acquisition age (Ellis, 1994). This study focused on linguistic distance between learners’ L1 and Chinese and Chinese proficiency level influences on Chinese learning. Future research could investigate the moderating effect of age on language knowledge acquisition under different linguistic distances and Chinese proficiency levels.^2^

## 9. Implications

To the best of our knowledge, this is the first empirical study to systematically examine the effect of linguistic distance between L1 and Chinese on Chinese character, vocabulary, and grammar knowledge acquisition by Chinese proficiency level, using HSK test data and a multidimensional quantitative linguistic distance method. The study findings have theoretical and practical implications.

From a theoretical perspective, first, this study provides additional systematic empirical evidence regarding the long-standing question about the role of learners’ LI in second language acquisition in cross-language transfer theory. Combined with linguistic distance’s varying degree of influence on each language knowledge for different Chinese proficiency levels, our findings fully reflect the complex and dynamic understanding of the cognitive and sociocultural schools of thought regarding the role of L1 in second language acquisition (Vygotsky, 1978; Ringbom, 2007). Second, our study also provides support for asymmetry in CSL learners’ Chinese character or morpheme writing and recognition development (Li et al., 2014; Zhang and Roberts, 2021). Third, this study not only provides empirical evidence for Chinese character, vocabulary, and grammar acquisition performance in CSL learners from the Sinosphere, but also provides a reference for the less-studied Chinese character, vocabulary, and grammar acquisition patterns in CSL learners from non-Sinosphere countries. Finally, our findings provide new insights into the role of CSL learners’ L1 in second language acquisition. L1 plays different roles in language skills and language knowledge acquisition, because the effects of L1 differences on language knowledge acquisition are more complex.

From a practical perspective, this study adds a new perspective to existing cross-language transfer studies that relied on specific feature comparisons to measure linguistic distance between L1 and Chinese. Second, our findings suggest that CSL teachers should pay more attention to developing learners’ Chinese character writing, and use comparative analysis methods to help learners notice minute grammatical differences. Third, we recommend that students be taught in separate classes based on whether they are from Sinosphere or non-Sinosphere countries, to improve overall teaching and learning efficiency.

## 10. Conclusion

Our study conducted three quasi-experiments to systematically examine the influence of linguistic distance on Chinese character, vocabulary, and grammar knowledge acquisition, and their developmental characteristics among CSL learners with different Chinese proficiency levels. For the Chinese character and vocabulary acquisition, linguistic distance’s effect on CSL learners with elementary, intermediate, and advanced Chinese proficiency levels was largely consistent with cross-language transfer theory, which assumes that shorter linguistic distance between L1 and Chinese facilitates acquisition. However, the effect of linguistic distance on CSL learners’ grammar acquisition at the elementary Chinese proficiency level was not significant, whereas the effect at intermediate and advanced proficiency levels showed a short-distance L1 interference effect. Further, as CSL learners’ Chinese language proficiency improved, the linguistic distance effect on Chinese character acquisition remained largely unchanged, while the effect on vocabulary acquisition gradually decreased and the effect on grammar acquisition gradually increased. The results show that linguistic distance has differential proficiency-dependent effects on Chinese character, vocabulary, and grammar acquisition.

## Data availability statement

The original contributions presented in the study are included in the article/supplementary material, further inquiries can be directed to the corresponding author.

## Author contributions

XC and JB conceived and designed the work. JB performed the statistical analysis and wrote the first draft of the manuscript. XC revised the manuscript critically. All authors contributed to manuscript revision, read, and approved the submitted version.

## Funding

This project was supported by the Key Projects of Beijing Language and Culture University Fund (Award Number 19ZDJ04).

## Conflict of interest

The authors declare that the research was conducted in the absence of any commercial or financial relationships that could be construed as a potential conflict of interest.

## Publisher’s note

All claims expressed in this article are solely those of the authors and do not necessarily represent those of their affiliated organizations, or those of the publisher, the editors and the reviewers. Any product that may be evaluated in this article, or claim that may be made by its manufacturer, is not guaranteed or endorsed by the publisher.

## Footnotes

^1^ https://wals.info

^2^We thank the editor for this recommendation.
